# Selenoprotein P Deficiency Is Associated with Early Signs of Kidney Disease and Hospitalization Risk in Heart Failure

**DOI:** 10.3390/nu18050721

**Published:** 2026-02-24

**Authors:** Marcus Andréas Ohlsson, John Molvin, Hannes Holm Isholth, Anders Christensson, Christopher Nilsson, Agne Laucyte-Cibulskiene, Anders Grubb, Lutz Schomburg, Amra Jujic, Martin Magnusson

**Affiliations:** 1 Department of Internal Medicine, Skåne University Hospital, 214 28 Malmö, Sweden; 2 Department of Clinical Sciences, Lund University, 205 02 Malmö, Swedenmartin.magnusson@med.lu.se (M.M.); 3 Department of Cardiology, Lund University, 205 02 Malmö, Sweden; 4 Department of Nephrology, Skåne University Hospital, 214 28 Malmö, Sweden; 5 Department of Clinical Chemistry, Skåne University Hospital, Lund University, 205 02 Lund, Sweden; 6 Institut für Experimentelle Endokrinologie, Campus Charité Mitte, Charite-Universitätsmedizin, 10115 Berlin, Germany; 7 Wallenberg Center for Molecular Medicine, Lund University, 205 02 Malmö, Sweden; 8 Hypertension in Africa Research Team (HART), North-West University Potchefstroom, Potchefstroom 2531, South Africa

**Keywords:** selenium, SELENOP, heart failure, kidney failure, chronic kidney disease, acute kidney injury, SGHS, selective glomerular hypofiltration syndrome

## Abstract

Selenium (Se), an essential trace element, is linked to poor prognosis in heart failure (HF) and kidney disease. Se deficiency (serum Se < 70 μg/L) has been associated with increased cardiovascular mortality. Selenoprotein P (SELENOP), the main Se transporter, reflects bioavailable Se. Selective glomerular hypofiltration syndrome (SGHS), defined by a cystatin C-based eGFR < 0.7 of creatinine-based eGFR, signals early kidney dysfunction and worsens HF outcomes. The prognostic role of SELENOP for SGHS and kidney-related hospitalization in HF remains unclear. Purpose: To assess whether SELENOP is associated with SGHS at baseline and future kidney disease hospitalization in acute HF patients. Methods: In 570 patients hospitalized for acute HF, creatinine and cystatin C were analyzed; SELENOP was measured in the first 320 using an immunoassay. Kidney hospitalizations (ICD-10 N17–N19) were identified from regional registries. Logistic and Cox regression models evaluated SELENOP’s association with SGHS and hospitalization risk, adjusting for age, sex, blood pressure, BMI, eGFR and NT-proBNP. Results: Among 320 patients (mean age 75 years, 69% male), 58% had Se deficiency, and 30% had SGHS. During a median 43-month follow-up, 28 patients were hospitalized for kidney disease. Higher SELENOP was linked to lower odds of SGHS (OR 0.69; *p* = 0.002) and reduced risk of hospitalization for AKI or CKD (HR 0.60; *p* = 0.010), particularly AKI (HR 0.42; *p* = 0.002). SELENOP-deficiency (<3.23 mg/L) predicted AKI hospitalization (HR 4.02; *p* = 0.035). Conclusions: Low SELENOP is associated with SGHS and increased risk of kidney disease hospitalization, especially AKI, suggesting Se status may influence HF and renal outcomes.

## 1. Introduction

Selenium (Se), a naturally occurring and essential trace element, was first discovered in 1817 by the Swedish chemist Berzelius [1]. Selenium’s pleiotropic properties have since been widely investigated and nowadays include antioxidant and anti-inflammatory properties [2,3,4]. Health benefits such as protection against cardiovascular disease, decreasing the incidence of cancer, strengthening the immune system, treatment for certain muscle disorders and improving quality of life in supplementation studies have been demonstrated [5,6,7,8].

The effect of Se deficiency on heart failure (HF) became evident in the 1930s, when a severe form of cardiomyopathy in Keshan County in China (Keshan disease) was discovered and several decades later linked to insufficient Se supply due to Se-deficient soils. A causal relationship between this specific type of cardiomyopathy and Se deficiency was established, and the condition was practically eradicated after Se supplementation was initiated [9,10]. Malnutrition of micronutrients, Se included, has been shown to affect mitochondrial function and contractility of cardiomyocytes [10]. Parallel with these findings, between 30 and 50% of patients with HF demonstrate an improper uptake of micronutrients [11,12]. A causative mechanism for Se deficiency and the onset of HF, however, remains unexplained [13].

Recently, Se has also been implicated in kidney disease. Many patients with either acute kidney injury (AKI) or chronic kidney disease (CKD) suffer from Se deficiency, and, for the latter group (CKD), this has been linked to both increased mortality and higher rates of rehospitalization [14,15,16,17].

Selenoprotein P (SELENOP), produced in the liver, is the major transporter of Se in the human body, safeguarding sufficient Se supply and availability in the central nervous system and the endocrine system [18]. SELENOP levels are closely linked to Se intake, and SELENOP has been shown to play a central role in transport, storage and recycling of Se, as it is taken up by target cells in a receptor-dependent manner. For this reason, SELENOP is considered to reflect the bioavailable fraction of Se in blood and the best biomarker of Se status in clinical studies [18,19,20,21,22]. Recent studies have highlighted the diagnostic value of SELENOP, as it correlated to mortality in a cross-sectional European population [23], and SELENOP deficiency indicated disease-related death risk in colorectal cancer patients [24]. An unbiased analysis of the plasma proteome from longitudinal UK Biobank samples revealed SELENOP as the key circulating mediator supporting high grip strength and longevity [25].

In the kidneys, SELENOP is taken up through the glomerular filtrate into the proximal tubule epithelial cells, a process mediated by the lipoprotein receptor megalin, as shown by Olson et al. [26].

Selective glomerular hypofiltration syndrome (SGHS), initially described as “shrunken pore syndrome”, was first described in 2015 by Grubb et al. [27]. SGHS is characterized by a significant difference in cystatin C-based estimated glomerular filtration rate (eGFR_cys_) and creatinine-based eGFR (eGFR_cr_), and is commonly defined as eGFR_cys_ < 70% or <60% of eGFR_cr_. The pathophysiological understanding is that the elimination of larger molecules (5–50 kDa), including cystatin C (~13 kDa), is impaired, whereas the elimination of smaller molecules such as creatinine (0.11 kDa) remains unaffected [28]. Previous studies have implicated SGHS in CKD and HF, as well as in increased overall mortality [28,29,30,31,32].

Despite accumulating evidence linking selenium deficiency to adverse cardiovascular and renal outcomes, it remains unclear whether SELENOP is associated with early glomerular filtration abnormalities such as SGHS or whether it predicts clinically relevant kidney-related hospitalizations in acute HF. We therefore hypothesized that lower SELENOP concentrations would be associated with prevalent SGHS and increased risk of subsequent kidney disease hospitalization.

## 2. Methods

### 2.1. Study Population

The HeARt and Brain Failure inVESTigation study in Malmö, Sweden (HARVEST-Malmö) is currently being conducted at Skåne University Hospital [33] and is a prospective observational study. Patients admitted to the Department of Internal Medicine or the Department of Cardiology for acute HF (ICD-10 I50-) are eligible to participate. Acute HF includes both de novo HF and decompensated chronic HF. The sole exclusion criterion is the inability to provide informed consent.

**Figure 1:**
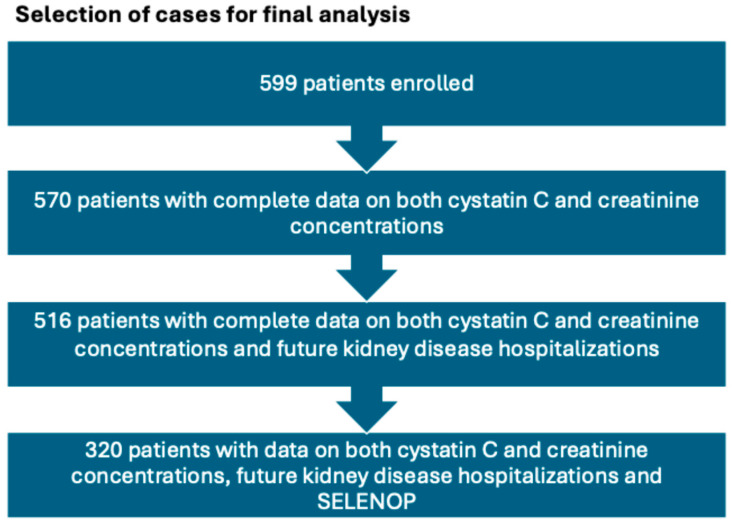
Selection of cases for the final analysis.

From March 2014 to March 2024, 599 patients were enrolled in the study. Among these, 570 had complete data on SGHS, and, of these patients, data concerning future hospitalizations for kidney disease was available in 516 cases. Baseline SELENOP measurements were available for the first 320 consecutive patients who were further examined in the present study. SELENOP measurements have not been performed in subjects recruited after this point. The selection of patients for the final analysis is illustrated in Figure 1.

The study was approved by the Ethical Review Board at Lund University, Sweden (Dnr 2013/360), and written informed consent was obtained from all participants.

### 2.2. Clinical Examination

Blood pressure was measured twice using a validated automated blood pressure monitor (Boso Medicus, Bosch and Sohn, Jungingen, Germany) after a 10 min rest, and the mean of the two readings was reported. Participants then underwent anthropometric measurements.

### 2.3. Definitions of Study Variables and Outcomes

Prevalent SGHS was defined as an eGFR_cys_/eGFR_cr_ ratio of less than 0.7 [34] in order to be able to identify milder forms of SGHS, indicating early signs of kidney dysfunction.

Body mass index (BMI) was calculated as weight in kilograms divided by height in meters squared (kg/m^2^). AKI was identified using ICD-10 codes N17.0–N17.9 retrieved from national registries, which include:

- N17.0: Acute kidney failure with tubular necrosis;
- N17.1: Acute kidney failure with acute cortical necrosis;
- N17.2: Acute kidney failure with medullary necrosis;
- N17.8: Other acute kidney failure;
- N17.9: Acute kidney failure, unspecified.

All kidney diseases, including both CKD and AKI, were defined using the aforementioned ICD-10 codes N17.0–N17.9, as well as N18–N19, which include:

- N18.1–5: Chronic kidney disease;
- N18.9: Chronic kidney disease, unspecified;
- N19: Unspecified kidney failure.

Se deficiency was defined as SELENOP < 3.23 mg/L, corresponding to approximately S-Se < 70 μg/L [35].

### 2.4. Laboratory Assays

Morning blood samples were collected upon admission and during the hospital stay after overnight fasting. Samples were collected, at the latest, within 72 h from admission. These samples were stored at −80 °C. Plasma cystatin C was analyzed at the Department of Clinical Chemistry, Skåne University Hospital, using an automated particle-based immunoassay, calibrated to the international reference preparation ERM-DA 471/IFCC (Hitachi Modular P analysis system; Roche, Basel, Switzerland). Plasma creatinine levels were measured using an enzymatic colorimetric assay with an IDMS-traceable calibrator on the same Hitachi Modular P analysis system (Roche, Basel, Switzerland). The revised Lund–Malmö (LMrev) equation was used to calculate eGFR_cr_ [36], while the Caucasian–Asian–Pediatric–Adult (CAPA) equation was used to calculate eGFR_cys_ [37]. The Chronic Kidney Disease Epidemiology Collaboration (CKD-EPI) equations were not used since the LMrev equation has previously been shown to outperform in a Swedish population [38]. In regression models, the combined eGFR_cr-cys_ was utilized and defined as the arithmetic mean of eGFR_cr_ and eGFR_cys_. SELENOP was analyzed in the first 320 consecutive subjects included in the study using a validated ELISA immunoassay (selenOtest, selenOmed GmbH, Berlin, Germany) utilizing monoclonal antibodies, as previously described elsewhere [39].

### 2.5. Primary Endpoint

- Presence of SGHS at baseline.

### 2.6. Secondary Endpoints

- Kidney disease hospitalization;
  ○ AKI or CKD;
  ○ AKI
- Description of associations between low SELENOP levels (<3.23 mg/L) and kidney disease hospitalizations.

### 2.7. Statistics

For normally distributed continuous variables, mean and standard deviation (SD) were used as descriptive measures, whereas median and interquartile range (IQR) were used for variables with non-normal distributions. Normality was assessed using visual inspection of histograms and skewness/kurtosis analysis. Student’s *t*-test or Mann–Whitney U-test was used to compare group means (medians) of continuous variables, and the Chi-square (χ^2^) test was used for comparison of group frequencies. Multivariable logistic, linear, and Cox regression analyses were adjusted for age, sex, systolic blood pressure, eGFR_cr-cys_ and BMI. Because of its relation to body composition and creatinine generation, BMI was included to account for potential confounding in the SGHS. For analyses investigating future hospitalization due to kidney disease, the multivariable model was also adjusted for NT-proBNP. Spearman correlation was used for correlation analyses. Values for highly sensitive C-reactive protein (hsCRP) and NT-proBNP were natural log-transformed prior to analysis to normalize their distribution. A Kaplan–Meier plot was used for cumulative hazard analysis. A two-sided *p*-value < 0.05 was considered as nominally statistically significant. All analyses were performed using SPSS statistical software version 30.0 for Windows (IBM Corp, released 2012, Armonk, NY, USA, IBM SPSS Statistics).

## 3. Results

### 3.1. Basic Characteristics

The study population characteristics are presented in Table 1. Of the total 320 patients with complete data on SELENOP, 187 (58%) patients fulfilled the criterion of Se deficiency. The mean age of the cohort was 75 years (±12.0), and 69% were male. The group with Se deficiency had significantly more patients with NYHA class III-IV, atrial fibrillation, and lower concentrations of creatinine. Presence of SGHS at baseline was more common among patients with Se deficiency; otherwise, no significant differences were seen between the groups. Subgroup analysis revealed no sex differences concerning the presence of SGHS (*p* = 0.368).

The median follow-up time was 43 months (IQR 14–81, range = 118).

### 3.2. Missing Data Analysis

A missing data analysis comparing patients with available SELENOP data (*n* = 320) to those without (*n* = 279) revealed a significantly higher proportion of patients with SGHS (*p* = 0.007) and NYHA III-IV (*p* = 0.008) in the group lacking SELENOP data. Otherwise, no significant differences were observed between the groups.

### 3.3. Primary Analyses

Logistic regression analysis showed a significant and strong inverse association between SELENOP and the presence of SGHS at baseline, as shown in Table 2.

Adjusted logistic regression analysis showed a non-significant association between SELENOP and the presence of CKD (eGFR < 60 mL/min/1.73 m^2^) at baseline (OR 1.23; 95% CI 0.97–1.57; *p* = 0.086). Correlation analyses yielded negative but non-significant results for SELENOP versus eGFR (*p* = 0.140) and cystatin C (*p* = 0.113), but a significant and positive correlation with creatinine (*p* = 0.010).

Multivariable linear regression analysis adjusted for SELENOP, hsCRP, age, sex and SBP showed a non-significant association between creatinine and SELENOP (β Coefficient 0.024; *p* = 0.457) at baseline. Further linear regression analysis of creatinine adjusted for low levels of SELENOP (SELENOP < 3.23 mg/L), hsCRP, age, sex, SBP and BMI yielded similar results (β Coefficient −0.061; *p* = 0.386). Furthermore, multivariable linear regression analysis adjusted for age, sex and SBP yielded a non-significant association between SELENOP and eGFR (β Coefficient −1.59; *p* = 0.064).

### 3.4. Secondary Analyses

Cox regression analysis showed a significant inverse association between SELENOP and future hospitalizations for kidney disease, as demonstrated in Table 3.

Using a cut-off of SELENOP < 3.23 mg/L as a proxy for Se deficiency, Cox regression analysis showed a robust and statistically significant association with hospitalizations for AKI, while the association with hospitalizations for AKI or CKD was attenuated, as shown in Table 4.

**Figure 2:**
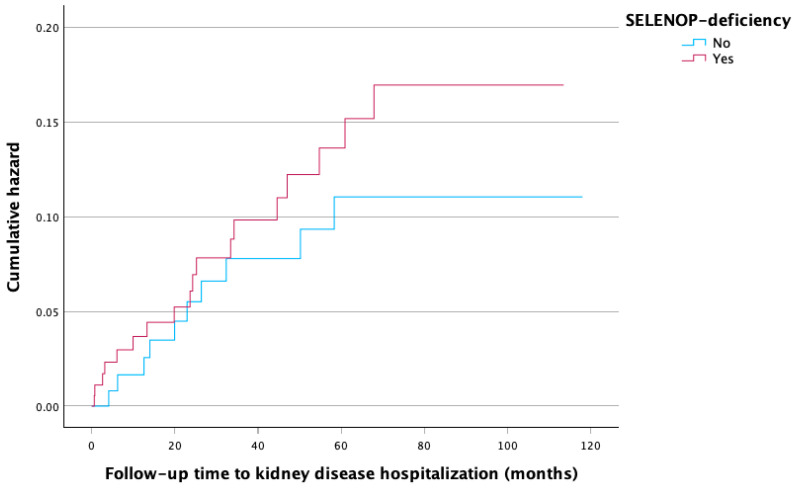
Kaplan–Meier plot illustrating the cumulative hazard of kidney disease hospitalization.

The cumulative incidence of kidney disease hospitalization stratified by SELENOP deficiency (<3.23 mg/L vs. ≥3.23 mg/L) is illustrated in Figure 2.

## 4. Discussion

In the current study, we characterized the serum SELENOP levels in patients admitted with acute HF and found that the majority had low SELENOP concentrations, corresponding to Se deficiency. Furthermore, we found that lower SELENOP concentrations were associated with the presence of SGHS as well as with a greater risk of future hospitalization for kidney disease.

These findings indicate an important role of selenoproteins in kidney disease in patients with acute heart failure. The association between SELENOP and SGHS may reflect the greater sensitivity of SGHS as a marker of kidney disease than the standard KDIGO guidelines, which do not identify all patients with SGHS [40,41]. Another possible reason for this association is that these patients may suffer from relative protein malnutrition, which is associated with both an increased incidence of SGHS [42] and Se deficiency [43,44].

Deterioration of kidney function among patients with HF is a serious complication that indicates advanced disease and is associated with poor prognosis [45]. Previous studies have shown that the concept of SGHS might add pertinent clinical information about kidney dysfunction that would otherwise not be detected by standard testing with plasma creatinine and/or eGFR alone [46]. The pathophysiological mechanisms behind the development of SGHS, however, remain unclear. So far, one histological study has reported morphological changes in the glomerular basal membrane in patients with SGHS and diabetes, showing that a thicker glomerular basal membrane correlated with a lower eGFR_cys_/eGFR_cr_ ratio [47].

In the present study, we demonstrate an association between low SELENOP and the presence of SGHS, but we make no claims regarding the potential causality of this finding, as the study design was observational. However, Se has previously been implicated in diabetic nephropathy, where Se supplementation significantly reduced levels of enzymes involved in the extracellular matrix degradation and decreased inflammation while improving protection from oxidative damage and total antioxidant capacity [48]. Furthermore, low Se levels have been observed in patients with CKD undergoing hemodialysis, and Se deficiency has also been associated with poor prognosis in this population [14,15,16]. In a study by Li et al., an association was found between low Se levels and a rapid decline in kidney function among hypertensive subjects [49]. Thus, there seems to be a direct connection between Se status and kidney function, which was further demonstrated in a small, yet randomized, study by Alehagen et al., where plasma creatinine levels decreased after 48 months of Se supplementation [50]. The mechanistic aspects of these associations are thought to involve the previously shown anti-inflammatory and anti-oxidative properties of Se-dependent selenoproteins.

Importantly, our results showed a very strong association of SELENOP deficiency, especially with future AKI hospitalization, suggesting that Se deficiency may increase susceptibility to AKI during episodes of hemodynamic instability or infection. This is biologically plausible, since selenoproteins such as glutathione peroxidases and thioredoxin reductases play key roles in counteracting oxidative stress and inflammation in glomeruli and tubuli [51,52], pathways central to both AKI and CKD progression.

Interestingly, the group with SELENOP deficiency demonstrated significantly lower creatinine concentrations, which was an unexpected finding. However, eGFR_cr_- and eGFR_cys_- levels between the groups did not display any significant differences, suggesting that the difference in creatinine is unlikely to reflect a true difference in kidney function. The underlying explanation is unclear; it might, however, be simply by chance, since the clinical significance of this finding is likely negligible. In order to elucidate this finding, we carried out additional linear regression analyses and found no significant associations between SELENOP and creatinine when adjusting for hsCRP, nor for low SELENOP concentrations and creatinine, even after adjustment for BMI to account for potential differences in muscle mass. Although additional multivariable analyses did not demonstrate an independent association between SELENOP and creatinine, the lower creatinine levels observed in the SELENOP-deficient group may reflect reduced muscle mass in patients with more advanced HF, rather than a true difference in renal function. This interpretation is supported by the higher prevalence of NYHA III-IV in this group and is consistent with the known association between HF severity, sarcopenia, and reduced creatinine generation. Alternatively, the higher prevalence of advanced heart failure (NYHA class III–IV) among SELENOP-deficient subjects could have contributed to lower creatinine values through congestion-related hemodilution.

## 5. Strengths

A key strength of this study is the use of a cohort that closely reflects a real-world clinical setting, enhancing the generalizability of the findings. The study also employs hard endpoints, including long-term kidney outcomes, which contribute to the robustness and reliability of the conclusions. Furthermore, the use of the eGFR equations LMrev and CAPA—both developed in a Swedish population and recognized by KDIGO as among the few validated formulas—adds methodological rigor and relevance to the analysis.

## 6. Limitations

This study has several limitations that should be acknowledged. The sample size for SELENOP is relatively small, and as a single-center investigation with a limited number of kidney disease hospitalizations, the findings may not be widely generalizable. Although low SELENOP remained significantly associated with future kidney-related hospitalization in the multivariable model, the estimate was imprecise, as reflected by wide confidence intervals.

A missing data analysis revealed a significantly higher proportion of patients with NYHA III-IV and SGHS in the group without SELENOP measurements; otherwise, no other significant differences were found. Because SELENOP data were less often available in sicker patients, the missing data may introduce a selection bias. We therefore acknowledge that the generalizability of our findings may be affected and recommend replication in cohorts with more complete biomarker data. Additionally, the cross-sectional observational design restricts our ability to infer causality. Moreover, potential confounding factors such as dietary intake, inflammatory status, and albumin levels were not accounted for in the data. Another limitation is that we lack data on muscle mass among our patients, which prevents us from further exploring what mediates the observation of lower creatinine levels among the SELENOP-deficient participants.

However, our findings demonstrate that reduced SELENOP concentrations are significantly associated with an increased risk of future hospitalization due to kidney disease. Kidney disease hospitalization represents a clinically more relevant outcome than subclinical changes in creatinine. To our knowledge, these findings are novel in the field and warrant further investigation into the relationship between kidney function and Se status, with potential implications for risk assessment and therapeutic strategies

## 7. Conclusions

In this study, we identified an inverse relationship between SELENOP and the presence of SGHS among patients admitted for acute heart failure, and showed that low SELENOP was associated with an increased risk of subsequent hospitalization for kidney disease. These findings indicate that assessment of selenium status could help identify HF patients at higher risk of kidney disease and guide preventive strategies such as nutritional optimization or supplementation. However, because of the observational design and selective biomarker availability, prospective interventional studies are needed before clinical implementation.

## Figures and Tables

**Table 1:** Basic characteristics of the study population.

Variable	Total	SELENOP < 3.23 mg/L	SELENOP ≥ 3.23 mg/L	*p*
	*n* = 320	*n* = 187	*n* = 133	
Age (years)	75 (±12)	75 (±11)	74 (±12)	0.666
Sex (male)	221 (69.1)	123 (65.8)	98 (73.7)	0.131
BMI (kg/m^2^)	27.8 (±5.9)	27.9 (±6.2)	27.7 (±5.3)	0.734
SBP (mmHg)	137 (±28)	135 (±27)	138 (±29)	0.888
RAAS inhibitors (*n*; %)	250 (78.1)	143 (76.5)	107 (80.5)	0.396
Diuretics (*n*; %)	313 (97.8)	183 (97.9)	130 (97.7)	0.944
MRA (*n*; %)	14 (4.4)	7 (3.7)	7 (5.3)	0.512
NYHA class III-IV (*n*; %)	273 (85.3)	168 (89.8)	105 (78.9)	0.007
Diabetes (*n*; %)	119 (37.2)	68 (36.4)	51 (38.3)	0.718
Atrial fibrillation (*n*; %)	152 (47.5)	98 (52.4)	54 (40.6)	0.030
SELENOP (mg/L)	3.10 (±1.12)	2.36 (±0.60)	4.16 (±0.79)	<0.001
NT-proBNP (ng/L)	4214 (2249–8887)	4557 (2465–10,178)	3820 (1921–7881)	0.092
Cystatin C (mg/L)	1.65 (1.33–2.13)	1.66 (1.34–2.22)	1.60 (1.30–2.07)	0.359
Creatinine (µmol/L)	105 (83–136)	100 (81–133)	114 (91–141)	0.012
eGFR *	51 (36–64)	52 (37–66)	49 (36–61)	0.077
eGFR_cys_	39 (28–52)	39 (26–50)	40 (29–53)	0.301
eGFR_crea_	51 (36–64)	52 (37–66)	49 (36–61)	0.089
KD hospitalization (*n*; %)	28 (8.8)	18 (9.6)	10 (7.5)	0.511
SGHS (*n*; %)	82 (25.6)	56 (29.9)	26 (19.5)	0.031

* eGFR based on the arithmetic mean of CAPA and LMrev formula combining creatinine and cystatin C. eGFR_cys_ is based on the CAPA-equation. eGFR_crea_ is based on the LMrev-equation. SGHS—selective glomerular hypofiltration syndrome; BMI—body mass index; SBP—systolic blood pressure; RAAS—Renin–Angiotensin–Aldosterone system; MRA—mineralocorticoid receptor antagonist; NYHA class—New York Heart Association Classification; SELENOP—Selenoprotein P; eGFR—estimated glomerular filtration rate; KF—kidney disease.

**Table 2:** Associations between SELENOP and SGHS at baseline.

	OR	95%CI	*p*
SELENOP (mg/L)	0.69	0.55, 0.87	0.002
Age (years)	1.08	1.05, 1.11	<0.001
Sex (female)	0.47	0.27, 0.83	0.009
SBP (mm/Hg)	1.01	1.00, 1.01	0.292
eGFR (mL/min/1.73 m^2^)	1.03	1.01, 1.04	<0.001
BMI (kg/m^2^)	1.02	0.98, 1.07	0.344

Values are odds ratios (ORs) and 95% confidence intervals. eGFR refers to the mean of CAPA (cystatin C) and LMrev (creatinine).

**Table 3:** Associations between SELENOP and future kidney disease hospitalization.

	AKI or CKD			AKI		
	HR	95%CI	*p*	HR	95%CI	*p*
SELENOP (mg/L)	0.60	(0.41, 0.89)	0.010	0.42	(0.24, 0.73)	0.002
Age (years)	0.96	(0.93, 0.99)	0.005	0.96	(0.92, 1.01)	0.102
Sex (female)	1.34	(0.59, 3.04)	0.484	1.45	(0.49, 4.34)	0.506
SBP (mmHg)	1.00	(0.98, 1.01)	0.625	1.00	(0.98, 1.02)	0.931
eGFR (mL/min/1.73 m^2^)	0.94	(0.92, 0.97)	<0.001	0.97	(0.94, 1.00)	0.083
NT-proBNP (ng/L)	1.22	(0.79, 1.87)	0.364	1.27	(0.71, 2.28)	0.426

Values are hazard ratios (HR) and 95% confidence intervals for hospitalization for AKI or CKD (ICD-10 codes N17–N19), or AKI (ICD-10 code N17). SBP—systolic blood pressure, eGFR refers to the mean of CAPA (cystatin C) and LMrev (creatinine).

**Table 4:** Associations between SELENOP < 3.23 mg/L and future hospitalization for kidney disease.

	AKI or CKD			AKI		
	HR	95%CI	*p*	HR	95%CI	*p*
SELENOP < 3.23 (mg/L)	1.77	(0.80, 3.89)	0.157	4.02	(1.10, 14.66)	0.035
Age (years)	0.96	(0.93, 0.99)	0.004	0.97	(0.93, 1.01)	0.096
Sex (female)	1.46	(0.65, 3.27)	0.389	1.52	(0.53, 4.39)	0.438
SBP (mmHg)	1.00	(0.98, 1.01)	0.560	1.00	(0.98, 1.02)	0.906
eGFR (mL/min/1.73 m)	0.95	(0.93, 0.97)	<0.001	0.97	(0.94, 1.01)	0.119
NT-proBNP (ng/L)	1.28	(0.83–1.97)	0.260	1.41	(0.79–2.52)	0.252

Values are hazard ratios (HRs) and 95% confidence intervals for hospitalization for AKI or CKD (ICD-10 codes N17–N19), or AKI (ICD-10 code N17). SBP—systolic blood pressure, eGFR refers to the mean of CAPA (cystatin C) and LMrev (creatinine).

## Data Availability

The original contributions presented in this study are included in the article. Further inquiries can be directed to the corresponding author.

## Abbreviations

SELENOP	Selenoprotein P
Se	Selenium
SGHS	Selective glomerular hypofiltration syndrome
CKD	Chronic kidney disease
AKI	Acute kidney injury
HF	Heart failure
